# Effects of the anti-inflammatory pentoxifylline on psychiatric and neuropsychiatric conditions: exploring various off-label utilities with meta-analyses

**DOI:** 10.1007/s10787-024-01616-7

**Published:** 2025-01-08

**Authors:** Ahmed Ramzi, Subhia Maya, Nadeen Balousha, Mufreh Amin, Robert Charles Powell, Mostafa Ramzi Shiha

**Affiliations:** 1 Faculty of Medicine, Mansoura University, Mansoura, Egypt; 2 Faculty of Medicine, Damascus University, Damascus, Syria; 3 Faculty of Pharmacy, Mansoura University, Mansoura, Egypt; 4 Faculty of Medicine, Misr University for Science and Technology, Giza, Egypt; 5 Boston NW Suburbs, USA; 6 Faculty of Urban and Regional Planning Cairo University, Giza, Egypt

**Keywords:** Pentoxifylline, Psychiatric symptoms, Neuropsychiatric symptoms, Major depressive disorder, Dementia, Meta-analysis

## Abstract

**Background:**

Chronic inflammation has been linked to many psychiatric disorders, and therefore, pertinent anti-inflammatory therapies have been empirically evaluated for management. An enduring example of long-term safety, attainability, and versatility has been pentoxifylline (PTX). PTX is a phosphodiesterase inhibitor that modulates inflammatory mediators and affects most blood components and the blood vessels.

**Methods:**

Major databases were systematically searched to identify randomized controlled trials (RCTs) on PTX in psychiatric and neuropsychiatric disorders until September 25, 2024.

**Results:**

21 RCTs were included. Five studies evaluated clinical depression: four on major depressive disorder (MDD) and one on bipolar patients experiencing treatment-resistant depression. PTX significantly reduced depressive symptoms in MDD in the four double-blind, randomized, placebo-controlled trials, with the three studies combining PTX and SSRIs showing statistically significant improvements in response rates. Ten RCTs on cognitive impairment reported beneficial effects, particularly in vascular dementia. Meta-analyses support its efficacy in reducing depressive symptoms, cognitive decline, asthenia, and inflammatory markers.

**Conclusion:**

Exploring the effects of PTX on psychiatric and neuropsychiatric conditions has provided considerable support for its utility across various disorders, most notably in moderate to severe major depressive disorder (as adjunctive therapy with SSRIs) and cognitive impairment in vascular dementia (as monotherapy). Relevantly, the potential of PTX across a wide range of conditions might prove beneficial in cases of co-occurrence.

**Supplementary Information:**

The online version contains supplementary material available at 10.1007/s10787-024-01616-7.

## Introduction

What is psychiatric and what is neurological are often interconnected, so we plan to examine both Psychiatric and Neuropsychiatric Symptoms as targets for the intervention at hand.

Based on global data, the prevalence and burden of psychiatric and neuropsychiatric conditions have been increasing in recent decades and are expected to continue rising, making the exploration of favorable interventions increasingly relevant (Global 2019; Collins et al. 2011; Wu et al. 1990).

Inflammation has been linked to many mental symptoms, and consequently, many anti-inflammatory interventions have been evaluated for their effectiveness and tolerance as add-on therapy in various mental health conditions (Dantzer et al. 2008; Chen et al. 2021; Fond et al. 2014; Nawras et al. 2023).

One such anti-inflammatory intervention is Pentoxifylline (PTX), a competitive type 3 and 4 phosphodiesterase inhibitor that influences various inflammatory mediators, making it a potential modulator for the inflammatory components of psychiatric and neuropsychiatric symptoms (Furth et al. 1997; Brie et al. 2016; Marques et al. 1999).

Its effects are not limited to anti-inflammatory or immune-modulatory actions but extend to affecting red blood cell deformability, most blood components, and the blood vessels. This opens yet another front with cognitive impairment, especially in vascular disorders and the geriatric population, which is another main topic addressed here (Samlaska and Winfield 1994; Aviado and Porter 1984; Rasyid et al. 2018).

Due to its versatility, effectiveness, cost-effectiveness, and safety profile, PTX may represent a promising candidate. It has indeed been evaluated in various pertinent clinical studies involving affective, psychotic, cognitive, neurotic, and behavioral conditions.

Therefore, the aim of this systematic review and meta-analysis is to evaluate the role of pentoxifylline in treating psychiatric and neuropsychiatric conditions. Specifically, we aim to appraise and synthesize evidence from randomized controlled trials to examine its effects on reported psychiatric manifestations, with a primary focus on depressive symptoms, cognitive impairment, and the safety of PTX regimens in these populations.

## Methods

We checked the Preferred Reporting Items for Systematic Reviews and Meta-Analyses (PRISMA) checklist and registered a protocol on the International Prospective Register of Systematic Reviews (PROSPERO) under ID CRD42024595249.

### Searches

For the main search, EMBASE, Scopus, PubMed, and Web of Science were last searched on 25 September 2024. The search included terms related to Pentoxifylline (Pentoxifylline, Trental, Oxpentifylline), combined with wildcard truncation to capture articles involving neurological, neuropsychiatric, psychiatric, cerebral, and mental disorder conditions (neuro*, psych*, cerebr*, menta*). Relevant site settings were used to trim the results, without any restriction on date, place, or language, with more details provided in the supplementary material.

For the introductory and supplementary searches, PubMed was used to systematically identify publication patterns of PTX, as well as to find clinical studies involving PTX active metabolites and close analogues (propentofylline [PPF], lisofylline [LSF], pentifylline, and CTP-499/PCS-499) to be employed in pertinent discussions.

### Study selection criteria

Study designs: Included are experimental randomized controlled trials (RCTs). Non-RCTs, observational studies, case reports, non-empirical studies, non-human or non-clinical research, and secondary research (reviews, systematic reviews, meta-analyses) are excluded.

Conditions and outcomes: Patients diagnosed with any psychiatric or neurological disorder that have neuropsychiatric symptoms evaluated as outcomes.

Intervention(s): Administration of pentoxifylline at any dosage or regimen, whether used alone or in conjunction with other therapies.

Comparator(s)/control: Placebo, absence of treatment, standard care, or other treatments.

### Study selection process

Duplicates are removed, followed by a two-step blinded screening by two independent reviewers: first, title and abstract screening to exclude ineligible studies; second, full-text screening to confirm eligibility for the systematic review. Conflicts are resolved through discussion or consultation with a third author if needed.

### Data extraction

Two researchers independently extract data from the included studies using a standardized data extraction form. That data extraction form includes these items: Study ID, study design, disease studied, sample size, age and gender %, intervention, comparator, treatment duration, primary outcomes and scales, secondary outcomes and scales, concise conclusion, country.

### Risk of bias (ROB)

ROB is independently evaluated by two authors using the Cochrane Collaboration's ROB2 tool for randomized trials, and robvis is employed for visualization.

### Synthesis

Thematically, studies are grouped by psychiatric or neuropsychiatric condition, and tables are created listing relevant studies and outcome data. We conduct a narrative synthesis by detecting recurring patterns, emphasizing significant findings, and contextualizing them within a broader view. Within each thematic area, we compare studies to identify consistencies, inconsistencies, discrepancies, and patterns, investigating potential sources of heterogeneity. The implications of the synthesized findings for clinical practice and future research are discussed, focusing on areas of agreement, remaining uncertainties, and the relevance of pentoxifylline in neuropsychiatry. All relevant outcome variables recurring in three or more studies are included in the quantitative analysis and subjected to statistical synthesis using RevMan software. For continuous variables of outcome measures, we use either the mean difference (MD) or the standardized mean difference (SMD), depending on the uniformity of the measurement scales across studies. The MD is used when the same scale is applied in all studies, while the SMD is employed for differing scales to standardize the results. For dichotomous outcomes, the risk ratio (RR) is calculated to compare the probability of an event between treatment and control groups. The choice between random-effects and fixed-effects models is based on the degree of heterogeneity, assessed using the *I*^2^ statistic. A random-effects model is applied if substantial heterogeneity (*I*^2^ > 50%) is detected, while a fixed-effects model is used if heterogeneity is relatively low (*I*^2^ ≤ 50%), assuming all studies estimate the same effect size, with variation due only to random error. All effect sizes are presented with 95% confidence intervals (CIs). Heterogeneity is also evaluated using the Chi^2^ test (Q test), with a *p*-value less than 0.10 indicating significant heterogeneity, and visual inspection of forest plots is performed to identify inconsistencies in effect estimates. Sensitivity analyses assess the robustness of the results by excluding high-risk bias studies, the studies with the highest contribution to heterogeneity or effect size, using different statistical models, and examining the impact of outliers. Where applicable, a subgroup analysis is conducted to compare the efficacy of the intervention as monotherapy versus adjunctive therapy.

## Results

### Introductory search results/publication pattern

Evaluating the evolution of pentoxifylline (PTX) research publications reveals distinct patterns that correlate with key regulatory milestones, namely FDA approval in 1984 and the drug's transition to off-patent status in 1997 (U.S. 2024; U.S. 2024).

PTX publications peaked in the early and mid-1990s, with a notable decline following its transition to generic status after the loss of patent protection. This suggests that regulation-related market changes likely had a substantial influence on clinical research activity.

This trend was particularly pronounced in PubMed's core clinical journals, reflecting broader interest in PTX beyond this collection of major American clinical research journals (United States National Library of Medicine 2020; Powell and Pentoxifylline 2015; Klein-Fedyshin and Ketchum 2023).

**Fig. 1:**
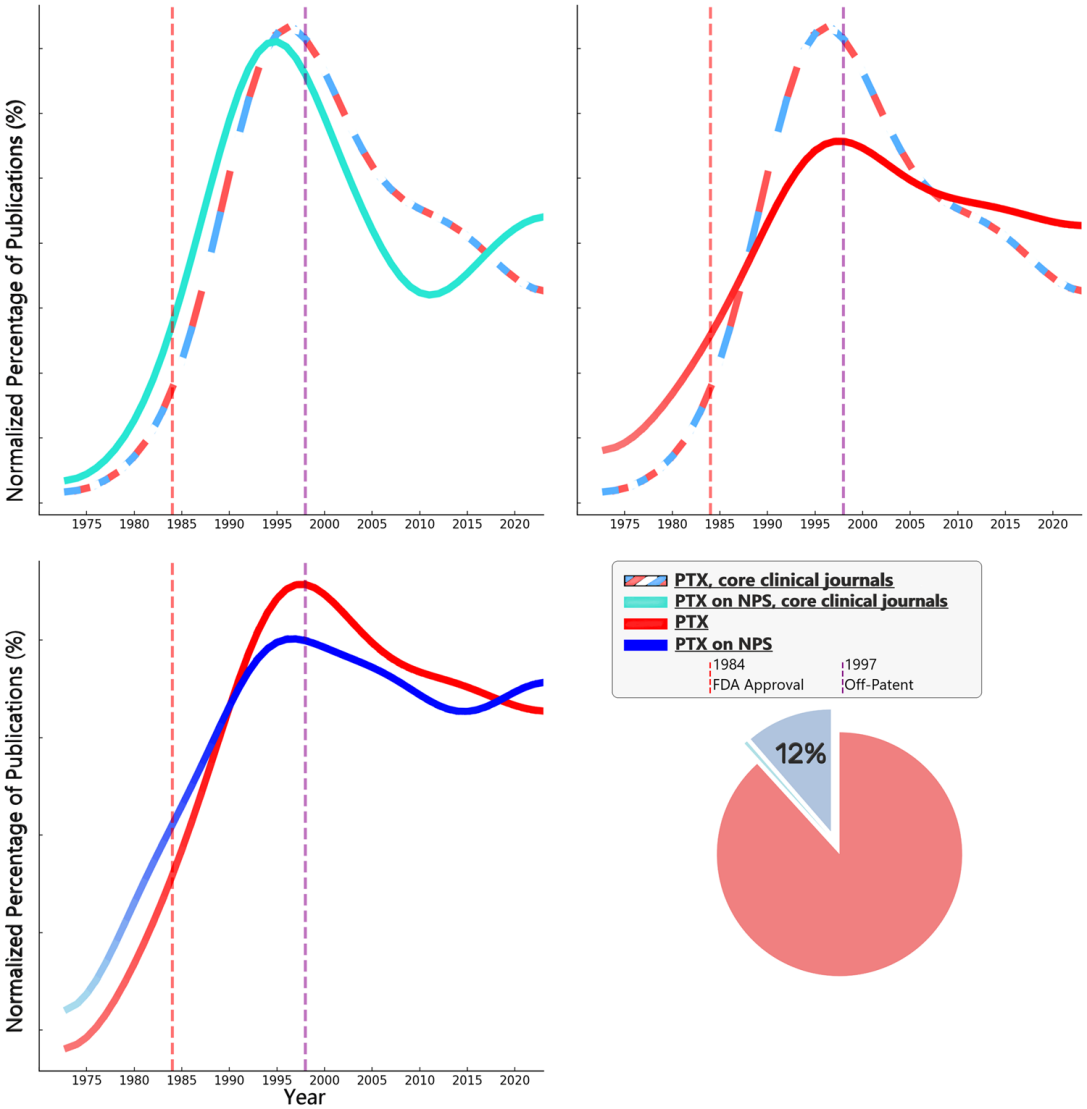
This compound figure consists of 4 sub-figures: three line graphs and one pie chart, each illustrating different aspects of publication patterns related to pentoxifylline (PTX) in human research on PubMed. Below is a detailed description: Top Left Graph (PTX General vs. NPS, in CCJ): Y-Axis: Normalized Percentage of Publications (%), X-Axis: Year (1973–2023).—Lines: Alternating Red-White-Blue: PTX publication trend in core clinical journals (CCJ). Turquoise line: PTX publication trend on neuropsychiatric subjects (NPS) within CCJ. Annotations: Vertical dashed red line: FDA approval of PTX (1984). Vertical dashed purple line: PTX off-patent year (1997). Top Right Graph (CCJ vs. All Journals): Axes and annotations: Same as above.—Lines: Alternating Red-White-Blue: PTX publication trend in CCJ. Solid Red: PTX publication trend across all journals. Bottom Left Graph (PTX General vs. NPS): Axes and annotations: Same as above.—Lines: Solid Red: PTX publication trend in general. Solid Blue: PTX publication trend on NPS. Pie Chart (Bottom Right): Proportion of PTX publications on NPS: Shaded bluish segment representing 12% of total publications

Publications regarding PTX on psychiatric and neuropsychiatric symptoms (NPS) followed a similar trajectory, peaking before 1997 but maintaining a relatively sustained proportion of overall PTX research over the years. Despite the decline in PTX studies after 1997, research on NPS has continued to account for around 11% of all PTX publications, indicating enduring interest in this domain. In recent years, a relative increase in research regarding PTX on NPS, possibly driven by emerging evidence, contributes to the rationale for this study. These patterns are visualized in a compound Figure **(**Fig. 1**).**

### Results of the main search

On 25 September 2024, a systematic updated search of four major electronic databases identified 5,607 studies: EMBASE (*n* = 711), PubMed (*n* = 1060), Scopus (*n* = 2953), and Web of Science (*n* = 883). After removing 331 duplicates and 4,911 records by search settings, 365 records were screened, resulting in 34 full-text articles assessed for eligibility. Ultimately, 21 randomized controlled trials (RCTs) were included in the review.

**Fig. 2:**
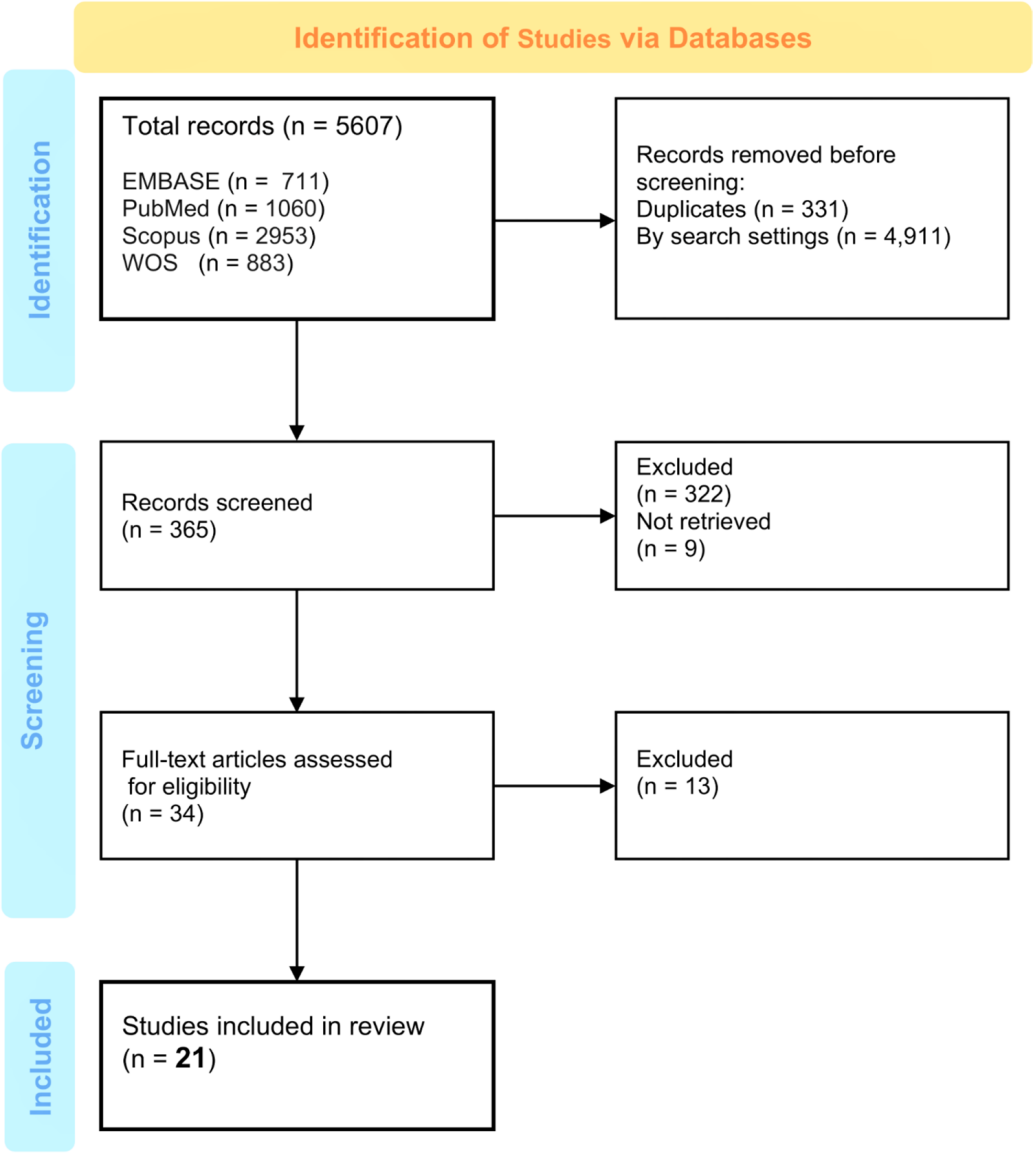
Curation

Curation is outlined in a flowchart (Fig. 2).

### Included studies

The list of the 21 included RCTs:

Merza Mohammad 2024 May (Merza Mohammad et al. 2024), August (Mohammad et al. 2024), Farajollahi 2021 (Farajollahi-Moghadam et al. 2021), (Yasrebi et al. 2021), (El-Haggar et al. 2018),

Sinichi (2023), Pavlov (2021), Al-Nimer (2019), Procházková (2018), Escolar (2012), Akhondzadeh (2010),

(1997), Bayer (EPMID) (1996), Black (1992), Blume (1992), Hartmann (1988), (Ghose 1987), Parnetti (1985), Parnetti (1984), Janaki (1980), Harwart (1979).

Theme-wise, studies will be addressed as follows: first we will introduce the studies on depression, mainly major depressive disorder (MDD), followed by the studies focusing on cognitive impairment (CI) and lastly a smaller group of neurodevelopmental and other neuropsychiatric conditions.

**Fig. 3:**
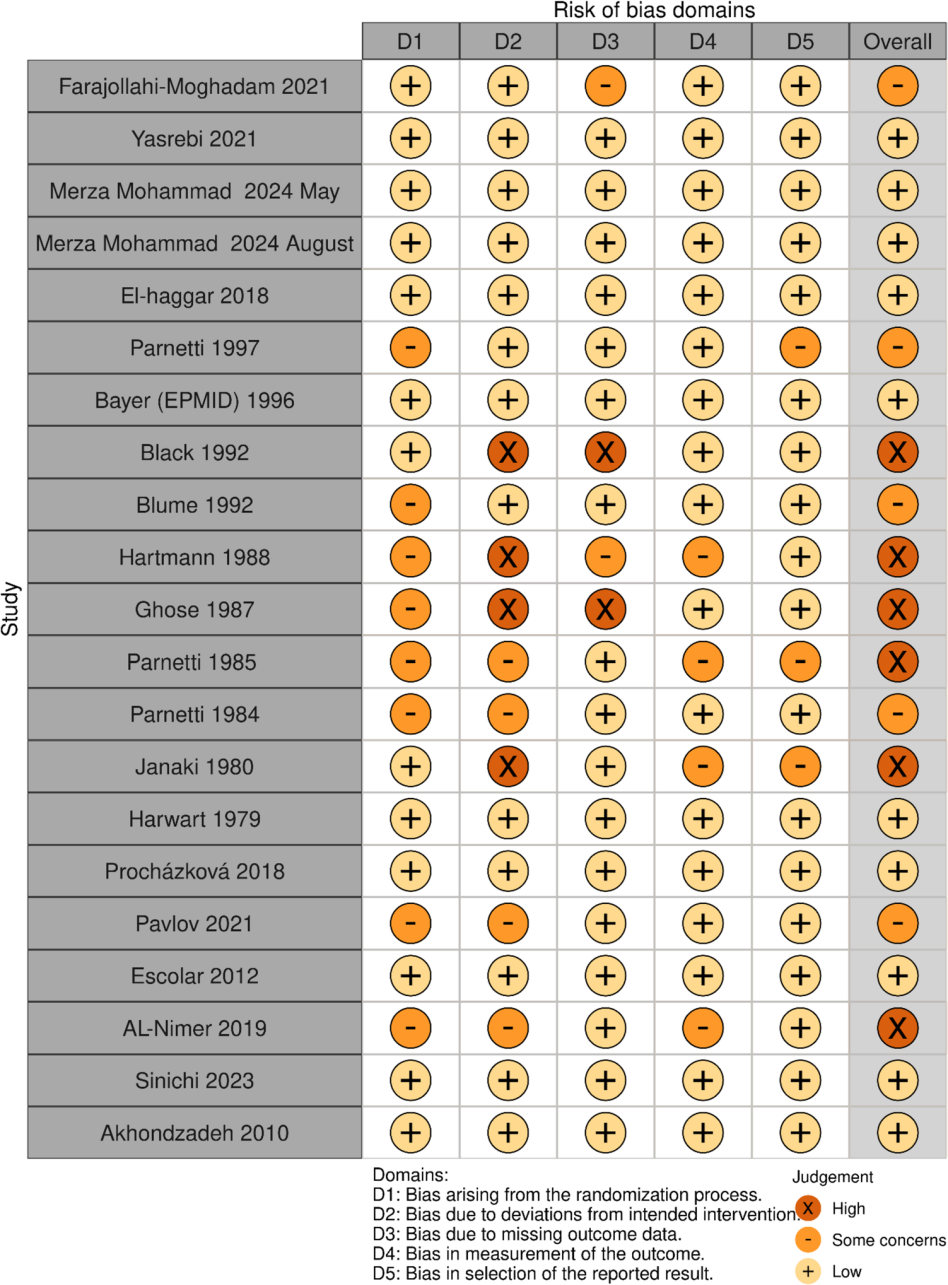
Risk of bias domains and judgment for the included RCTs, using the ROB2 tool

Risk of bias (ROB) appraisal:

Visualized Quality/ ROB assessment for 21 RCTs included in the study is provided in the following Fig. 3. It is notable that approximately half of the studies were assessed to have a moderate or high risk of bias, mainly in the older studies pertaining to cognitive impairment.

### Studies in affective disorders

Five randomized, double-blind, placebo-controlled trials used PTX versus placebo for treating depression in 360 patients (Merza Mohammad et al. 2024; Mohammad et al. 2024; Farajollahi-Moghadam et al. 2021; Yasrebi et al. 2021; El-Haggar et al. 2018). Four focused on major depressive disorder (MDD), with two investigating moderate depression (El-Haggar 2018, Yasrebi 2021) and two examining severe depression (Farajollahi 2021, Merza Mohammad May 2024), while one study (Merza Mohammad August 2024) addressed bipolar I/II with treatment-resistant depression (TRD), classified as severe depression.

The Hamilton Rating Scale for Depression (HAM-D-17) was used to assess depressive symptoms, applied as an inclusion criterion in MDD, and the response rate was uniformly defined as a ≥ 50% drop in HAM-D-17 scores.

Regarding MDD, in three of the four studies, PTX was used as an adjuvant to a selective serotonin reuptake inhibitor (SSRI). However, even the one study that used PTX as a sole intervention also reported a significant reduction in depressive symptoms compared to placebo, but—unlike the other studies—it did not achieve a significant intergroup difference in response rates. None of the studies showed a significant difference between PTX and placebo in terms of side effects.

The last and latest study focused not on MDD but on bipolar I/II patients with severe, treatment-resistant depression (TRD), using PTX as a sole intervention. In this 12-week RCT, no significant overall difference in HAM-D-17 score reduction was observed between PTX and placebo. Response rates were not significantly different between groups overall, but were higher for PTX in the high-CRP subgroup and lower in the low-CRP subgroup. PTX significantly reduced CRP, TNF-α, and IL-6 within its group, while the placebo group showed no significant reductions. Intergroup, only CRP showed a significant difference. The researchers suggested that PTX may be more effective in patients with elevated inflammatory status due to its strong anti-inflammatory effects.

**Fig. 4:**
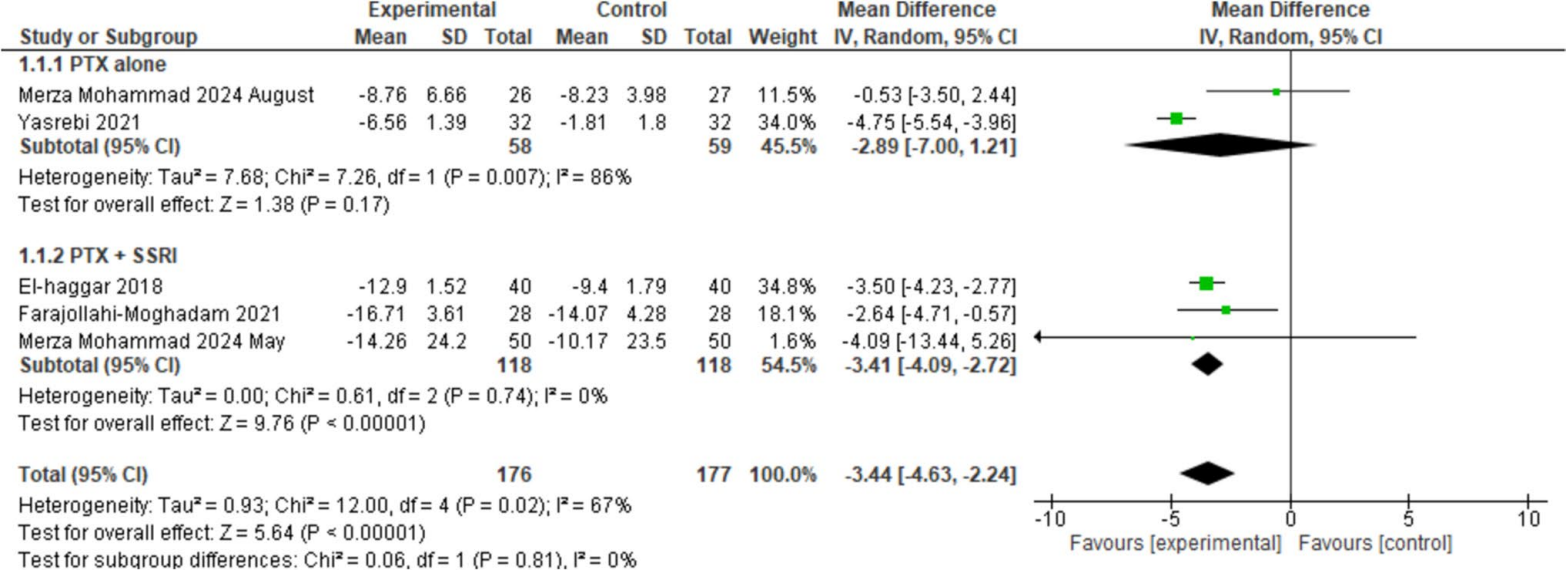
The meta-analysis evaluates the mean difference in HAMD score reductions in patients with depression treated with Pentoxifylline (PTX) alone or combined with Selective Serotonin Reuptake Inhibitors (SSRI). Two studies on PTX alone (Merza Mohammad 2024 August, Yasrebi 2021) show a pooled mean difference of − 2.89 [95% CI (− 7.00, 1.21), *p* = 0.17] with significant heterogeneity (I^2^ = 86%). Three studies on PTX + SSRI (El-Haggar 2018, Farajollahi-Moghadam 2021, Merza Mohammad 2024 May) show a pooled mean difference of − 3.41 [95% CI (− 4.09, − 2.72), *p* < 0.00001] with no heterogeneity (*I*^2^ = 0%). The overall pooled mean difference across all studies is − 3.44 [95% CI (− 4.63, − 2.24), *p* < 0.00001] with moderate heterogeneity *I*^2^ = 67%

**Fig. 5:**
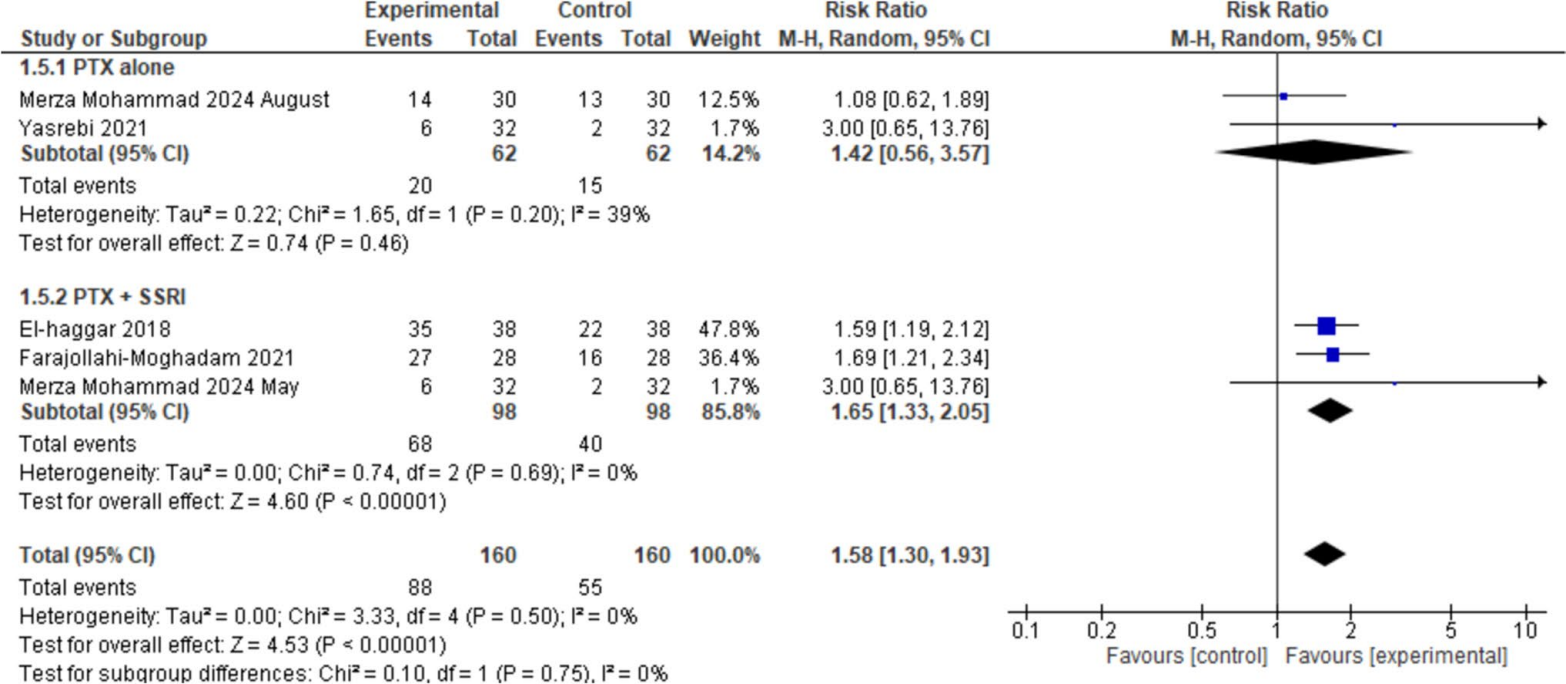
Response rate in depression with Pentoxifylline (PTX) alone or combined with Selective Serotonin Reuptake Inhibitors (SSRI). Two studies assessing PTX alone (Merza Mohammad 2024 August, Yasrebi 2021) show no significant effect with a pooled risk ratio (RR) of 1.42 [95% CI (0.56, 3.57), *p* = 0.46] and no heterogeneity (*I*^2^ = 0%). In contrast, three studies (El-Haggar 2018, Farajollahi-Moghadam 2021, Merza Mohammad 2024 May) evaluating PTX + SSRI show a significant increase in response rate with a pooled RR of 1.65 [95% CI (1.33, 2.05), *p* < 0.00001] and no heterogeneity (*I*^2^ = 0%). Overall, the pooled RR across all studies is 1.58 [95% CI (1.30, 1.93), *p* < 0.00001] with no significant heterogeneity (*I*^2^ = 0%)

**Fig. 6:**
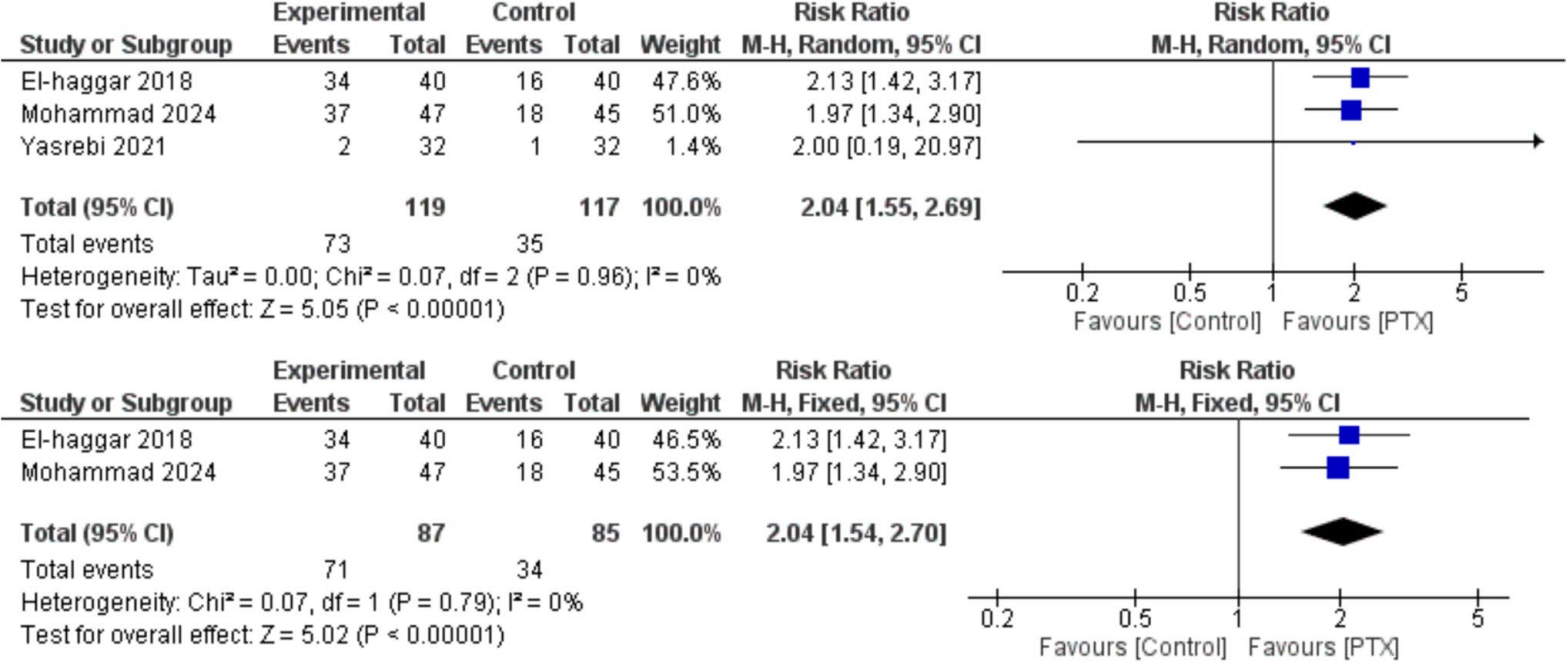
Remission rate in MDD patients was evaluated through a meta-analysis of 3 studies (236 participants total). The synthesized risk ratio (RR) was 2.04 (95% CI 1.55–2.69, *p* < 0.00001), showing a significantly higher remission rate in the Pentoxifylline group compared to the control, with no heterogeneity detected (*I*^2^ = 0%). The subsequent forest plot displays a sensitivity analysis that excluded the Yasrebi 2021 study, which had minimal influence on the overall analysis. This adjustment did not impact the significance of the results, as the pooled risk ratio remained at 2.04 (95% CI 1.54–2.70, *p* < 0.00001) with zero heterogeneity (*I*^2^ = 0%)

Characteristics and summaries of these studies are presented here in Table 1 in a concise form, and in an extended Table in the supplementary file. Figures 4, 5, and 6 present a statistical synthesis of these studies regarding HAM-D score reduction, response rate, and remission rate.

**Table 1:** Concise presentation of depression RCTs

IDs	N	condition	HAM-D-17 score -Inclusion criterion -Score at baseline PTX, PBO -Degree of depression (mild,mod,severe)	Experimental	PTX (mg/d)	W	Control	Response % (N/Total)
Farajollahi 2021	28:28	MDD	> 18 26.07 ± 3.45 25.82 ± 3 (severe)	SSRI + PTX	1200	6	SSRI + PBO	PTX: 96% Control: 57%
El-haggar 2018	40:40	MDD	≥ 18 19.85 ± 1.39 19.38 ± 1.25 (moderate)	SSRI + PTX	800	12	SSRI + PBO	PTX: 92% Control: 58%
Merza Moh. 2024 May	50:50	MDD	≥ 18 27.6 ± 9 26.1 ± 1.9 (severe)	SSRI + PTX	800	12	SSRI + PBO	PTX: 83% Control: 49%
Yasrebi 2021	32:32	MDD	(HAM-D = 14–17) 15.97 ± 0.9 15.72 ± 1.1 (Mild/mod)	PTX (alone)	800	6	PBO (alone)	W2, 4: PTX and PBO 0, W6: PTX 15%, PBO 3%
Merza Moh. 2024 August	30:30	Bipolar I/II with TRD	TRD 28.74 ± 5.6 29.59 ± 6.6 (severe)	PTX (alone)	800	12	PBO (alone)	PTX: 46.6%, PBO: 43.4%
Abbreviations	N: Number, W: Weeks, d: day, PBO: Placebo

### Studies pertaining to cognitive impairment

Older studies conducted in older populations evaluated the efficacy of pentoxifylline (PTX) in mitigating cognitive deterioration across various neurocognitive and cerebrovascular conditions, with sample sizes ranging from approximately 40 to 290 participants. All ten of these studies were randomized controlled trials (RCTs), with the majority being placebo-controlled and most reported following double-blind methodologies, although some varied in design (Parnetti et al. 1997, 1985; Bayer 1996; Black et al. 1992; Blume et al. 1992; Hartmann and Tsuda 1988; Ghose 1987; Janaki 1980; Harwart 1979).

The usual dose of pentoxifylline, 400 mg tablets taken three times daily, was used in almost all of the studies. The outcomes evaluated included cognitive function assessments, mental state evaluations, and clinical symptoms relevant to cerebrovascular insufficiency and dementia. Scales such as the Mini-Mental State Examination (MMSE) and the Sandoz Clinical Assessment-Geriatric (SCAG) were commonly used to quantify these outcomes.

Significant improvements in cognitive and clinical parameters were reported in most studies, with PTX consistently showing a beneficial effect compared to placebo or other comparators. Generally, no significant differences in side effects were reported between the PTX and placebo groups, indicating a favorable safety profile for PTX.

**Fig. 7:**
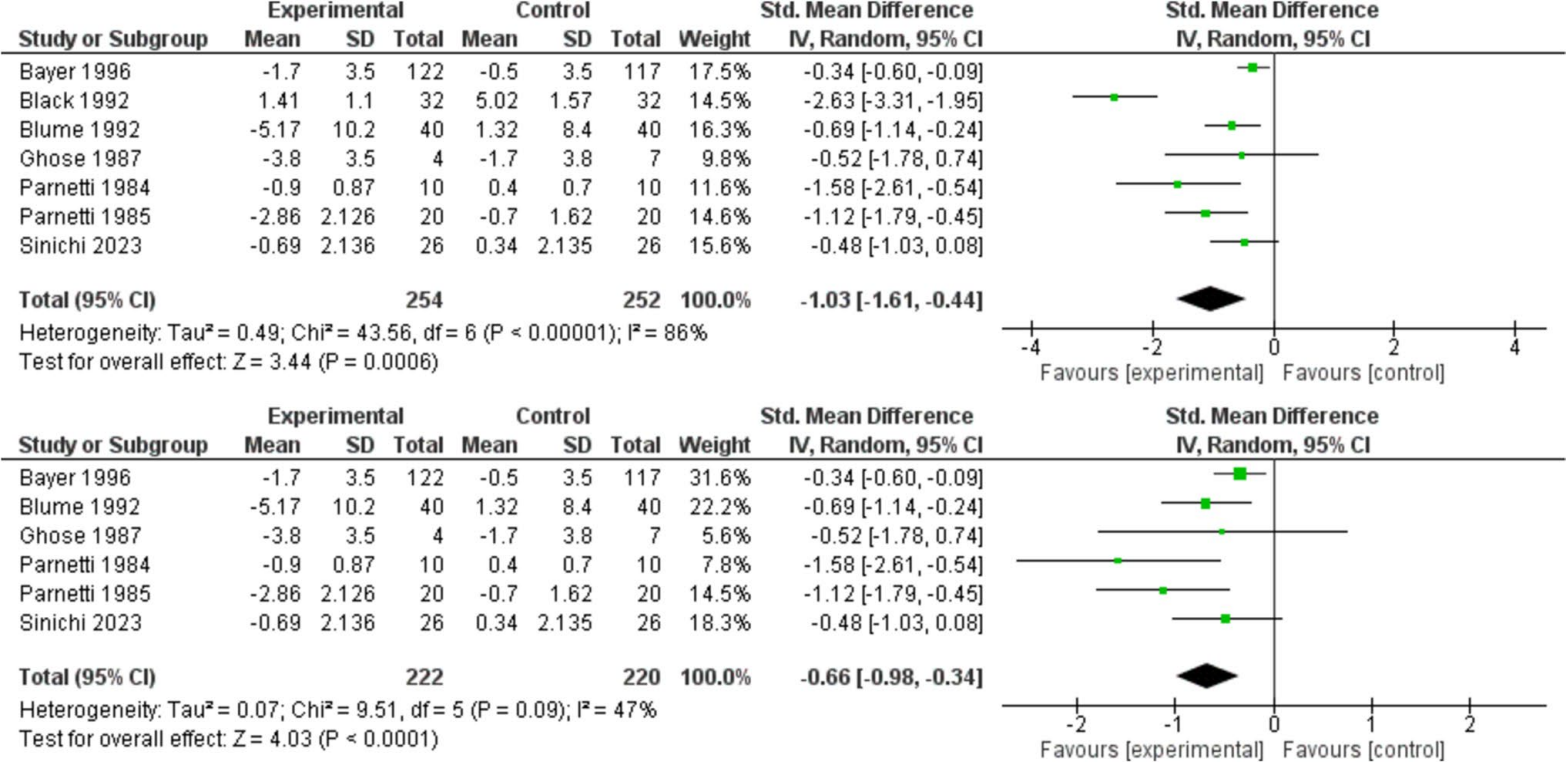
This meta-analysis evaluates the mitigation of cognitive deterioration across 7 studies (totaling 506 participants). The pooled standardized mean difference (SMD) was − 1.03 (95% CI − 1.61 to − 0.44, *p* = 0.0006), indicating a significant reduction in cognitive decline in the experimental group (Pentoxifylline) compared to the control (placebo). The analysis revealed high heterogeneity (*I*^2^ = 86%). The second part of the figure presents a sensitivity analysis that excluded the study by Black 1992, which was identified as a major source of heterogeneity. After this exclusion, the results remained significant, with a pooled SMD of − 0.66 (95% CI − 0.98 to − 0.34, *p* < 0.0001), and heterogeneity was substantially reduced (*I*^2^ = 47%)

Detailed characteristics and outcomes of these studies are presented in Table 2, with extended data available in the supplementary file. Quantifiable results pertaining to cognitive impairment are meta-analyzed in Fig. 7.

**Table 2:** Characteristics of studies on cognitive impairment

10 IDs	Design (All RCTs)	Disease studied	Sample size	Age (mean ± SD) Female Gender %	PTX Role	PTX mg/d	W	Conclusion
Hartman 1988	Open-label no placebo (3 groups)	Chronic Cerebro- vascular disease	Total: 90 PTX + BT: 30 EA + BT: 30 BT:30	most over 60 Fem. 43%	solo	1200	8	PTX significantly increased rCBF and improved symptoms such as sleep disturbances, vertigo, and tinnitus. EA also increased rCBF but was less effective than PTX
Harwart 1979	Double- blind, Placebo- controlled	Chronic Cerebro- vascular insufficiency	Total: 60 PTX: 30 PBO: 30	PTX: 78 ± 14 Fem. 83% PBO: 79 ± 7 Fem. 83%	solo	1200	8	PTX significantly improved clinical symptoms and psychometric test performance compared to placebo, with no major differences in laboratory parameters and side effects
Bayer (EPMID) 1996	Double- blind, placebo- controlled, multicentre	Multi-infarct dementia	Total: 289 PTX: 122 PBO: 117	PTX: 70 ± 9, Fem. 41% PBO: 69 ± 9, Fem. 47%	solo	1200	39	Treatment with PTX is beneficial for patients with Multi-infarct disease, the global results of the GBS and SCAG scales enforced significant improvements in subscales specific to intellectual and cognitive function
Parnetti 1985	Open-label placebo- controlled (4 groups)	Initial mental deterioration	Total: 80 PTX: 20 PBO: 20	PTX: 73 ± 1, Fem. 45% PBO: 72 ± 1, Fem. 45%	Both	1200	28	PTX enhanced red cell deformability but didn't affect cognitive tests alone. Combined with piracetam, it improved both cognitive performance and deformability beyond piracetam or placebo, showing synergy
Parnetti 1984	Double- blind Placebo- controlled (4 groups)	Primary Initial mental deterioration	Total: 40 PTX: 10 PBO: 10	PTX: 68 ± 3, Fem. 50% PBO: 71 ± 6, Fem. 60%	solo	1200	12	PTX showed significant improvements in psychometric test scores, in addition to improvements in hemorheological parameters
Black 1992	Double- blind placebo- controlled (4 groups)	Vascular dementia	Total: 64 PTX: 32 PBO: 32	PTX: 75 ± 10 Fem. 56% PBO: 76 ± 10 Fem. 41%	solo	1200	36	PTX significantly slowed cognitive deterioration as assessed by ADAS
Parnetti 1997	Double- blind double- dummy	Vascular dementia	Total: 93 PTX: 44 sdx: 49	PTX: 76 ± 4, Fem. 66% sdx: 75 ± 4, Fem. 55%	solo	1200	24	Both PTX and Sulodexide showed a statistically significant reduction in fibrinogen levels. However, GBS scores showed a significant improvement only in the sulodexide -not PTX- group
Blume 1992	Double- blind placebo- controlled	Vascular dementia	Total: 80 PTX: 40 PBO: 40	mean 64 (55–75) Fem. 40%	solo	1200	24	Patients treated with PTX showed statistically significant cognitive improvements compared to the placebo group
Janaki 1980	Open-label no placebo	Cerebral vascular accidents (thrombosis)	Total: 58 PTX: 10 X: 8	Most over 40 Fem. 31% & 41%	solo	600	8	Only patients treated with PTX showed a significant increase in their post-treatment scores from week 2 onwards
Ghose 1987	Double- blind Placebo- controlled	Multi-infarct dementia	Total: 28 PTX: 12 PBO:16	PTX: 76 ± 9, Fem. 50% PBO: 77 ± 5, Fem. 50%	Both	600- 1200	12	PTX administration resulted in a greater improvement in FMMS scores in MID patients compared to placebo, but this improvement was not observed in PDD patients

*PTX* pentoxifylline, *PBO* Placebo, *W* Weeks, *Fem* Females, *mg/d* milligrams/day, *RCTs* Randomized controlled trials. *BT* basic therapy, *EA* Ergot Alkaloid, *sdx* Sulodexide, *x* Xanthinol nicotinate, *rCBF* Regional cerebral blood flow, *GBS* scale, Gottfries, Brane, Steen scale, *SCAG* Sandoz Clinical Assessment Geriatric scale, *ADAS* Alzheimer’s Disease Assessment Scale, *FMMS* Folstein's Mini-Mental State, *PDD* Primary degenerative dementia, *MID* Multi-infarct dementia

### Studies on neurodevelopmental and other neuropsychiatric conditions

Studies of PTX in the domain of neuropsychiatry are not limited to depression and cognitive impairment but extend to neurodevelopmental disorders such as autism and schizophrenia, as well as other neuropsychiatric conditions like asthenia, anxiety, and NPS-related quality of life (QOL) (Sinichi et al. 2023; Pavlov et al. 2021; Al-Nimer et al. 2019; Procházková et al. 2018; Escolar et al. 2012; Akhondzadeh et al. 2010).

In almost all of these studies, PTX induced notable improvements.  The characteristics of the studies are summarized in Table 3.

**Table 3:** Brief description of studies on neurodevelopmental and other neuropsychiatric conditions

6 IDs	Design (All RCTs)	Disease Studied	Sample size	Age (mean ± SD) Female Gender %	PTX Role	PTX mg/d	W	Conclusion
Sinichi 2023	Double- blind placebo- controlled	Schizophrenia	Total: 52 PTX: 26 PBO: 26	PTX: 38 ± 11 Fem. 23% PBO: 41 ± 9 Fem. 42%	Add-on	800	8	PTX with risperidone improved cognitive impairments and positive schizophrenia symptoms, with no serious side effects, enhancing antipsychotic effects
Akhondzadeh 2010	Double- blind placebo- controlled	Autism	Total: 40 PTX: 20 PBO: 20	PTX: 8 ± 2 Fem. 20% PBO: 7 ± 2 Fem. 25%	Add-on	600	10	PTX with risperidone significantly improved behavioral symptoms in children with autism, without serious side effects
Procházková 2018	Double- blind, Double- dummy	Chronic Tinnitus (with related NPS)	Total: 197 EGb: 99 PTX: 98	PTX: 53 ± 11 EGb: 55 ± 11 Fem. 60%	solo	1200	12	Both treatments significantly improved Mini-TQ scores, tinnitus loudness, annoyance, anxiety (HADS), and illness-related disability. EGb 761® had fewer adverse events
Pavlov 2021	Open-label no placebo	Severe asthenic syndrome in NAFLD	Total: 247 Main: 124 Cont: 123	Main: 54 ± 3 Cont:54 ± 4 Fem. 0%	Add-on	1200	8	Early inclusion of PTX and Cytoflavin in the treatment regimen for NAFLD with severe asthenic syndrome significantly improved clinical, laboratory, and neuropsychological outcomes
Al-Nimer 2019	Single- blind Placebo- controlled	Diabetic Foot Syndrome (DFS)	PTX: 20 PBO: 20	56 ± 8, Fem. 70%	Add-on	1200	8	PTX significantly improved quality of life in DFS patients, particularly in emotional, social, and physical health, and reduced fatigue, as shown by SF-36 subscales
Escolar 2012	Double- blind, placebo- controlled	Duchenne Muscular Dystrophy (DMD)	Total: 64 boys PTX: 30 PBO: 32	PTX: 9.9 ± 2.9 PBO: 10.2 ± 2.8 Fem. 0%	Add-on	400- 1200	52	PTX, combined with corticosteroids, did not significantly improve muscle strength compared to placebo. Most outcomes, including PedsQL and its neuropsychiatric items, showed no significant differences

*PTX* pentoxifylline, *PBO* Placebo, *W* Weeks, *Fem* Females, *mg/d* milligrams/day, *Mini-TQ* psychological burden of tinnitus, *RCTs* Randomized controlled trials

### Meta-analyses

Further meta-analysis is in the supplementary.

## Discussion

Despite the relative decline in Pentoxifylline (PTX) research after it went off-patent, sustained interest in its neuropsychiatric applications has endured and been rejuvenated by recent evidence, reinforcing the rationale for systematically synthesizing existing data, including through meta-analyses.

### Clinical findings, limitations, and recommended research

Pentoxifylline (PTX) shows significant potential across a wide range.

In depression, PTX demonstrates significant efficacy in treating major depressive disorder (MDD) when used adjunctively with selective serotonin reuptake inhibitors (SSRIs) at doses of 800–1200 mg/day. In severe MDD, adjunctive PTX leads to greater reductions in HAM-D-17 scores and higher response rates compared to SSRIs with placebo. PTX monotherapy at 800 mg/day is less effective; in moderate MDD, it reduces symptoms without significantly improving response rates, and in treatment-resistant bipolar depression, it benefits patients with elevated inflammatory markers. These results indicate that PTX's effectiveness may depend on depression severity, dosing, and its role as an adjunct rather than monotherapy. Pertinently, the studies are relatively recent and are assessed to have high rigor, owing to ROB2 evaluations and their design as double-blind, placebo-controlled RCTs.

In cognitive impairment, PTX improved function in cerebrovascular diseases and dementia, as measured by scales such as SCAG and MMSE, with part of that effect probably attributed to enhanced cerebral blood flow. However, some studies found no significant effect, but the overall pooled effect was significant. This was assessed using SMD and a random-effects model to account for variability in related scales.

In most studies on cognitive impairment, it was used as monotherapy, and in most of the other studies, as an add-on. Individual RCTs showed disease-specific efficacy in schizophrenia, autism, tinnitus, and asthenic syndrome, with consistent safety and tolerability. Undeterred by the expected heterogeneity, small sample sizes, and short study duration in multiple instances, PTX's broad applicability and favorable safety profile support its potential in a variety of neuropsychiatric conditions. These results, though preliminary, may indicate promising potential and valuable versatility.

For each of the conditions addressed, future standardized trials would be valuable to better substantiate the findings and reinforce existing results. Especially since it is a widely well-tolerated, safe, and globally attainable medication, further large-scale empirical research is recommended to explore each domain further, including the investigation of alternative doses and regimens for enhanced therapeutic outcomes, ultimately advancing the evidence.

## Conclusion

Exploring the effects of PTX on psychiatric and neuropsychiatric conditions has provided considerable support for its utility across various disorders, most notably in moderate to severe major depressive disorder (as adjunctive therapy with SSRIs) and cognitive impairment in vascular dementia (as monotherapy).

Reports of efficacy also extended to neurodevelopmental disorders such as autism and schizophrenia, as well as NPS-related quality of life factors, all with a favorable safety profile. Relevantly, the potential of PTX across a wide range of conditions might prove beneficial in cases of co-occurrence.

## Supplementary Information

Below is the link to the electronic supplementary material.

Supplementary file1 (XLSX 231 KB)

## Data Availability

Enquiries about data availability should be directed to the authors.
